# Extensive Gene-Specific Translational Reprogramming in a Model of B Cell Differentiation and Abl-Dependent Transformation

**DOI:** 10.1371/journal.pone.0037108

**Published:** 2012-05-31

**Authors:** Jamie G. Bates, Julia Salzman, Damon May, Patty B. Garcia, Gregory J. Hogan, Martin McIntosh, Mark S. Schlissel, Pat O. Brown

**Affiliations:** 1 Department of Biochemistry, Stanford University School of Medicine, Stanford, California, United States of America; 2 Howard Hughes Medical Institute, Stanford, California, United States of America; 3 Computational Proteomics Laboratory, Fred Hutchinson Cancer Research Center, Seattle, Washington, United States of America; 4 Department of Molecular & Cell Biology, University of California, Berkeley, California, United States of America; University of Miami, United States of America

## Abstract

To what extent might the regulation of translation contribute to differentiation programs, or to the molecular pathogenesis of cancer? Pre-B cells transformed with the viral oncogene v-Abl are suspended in an immortalized, cycling state that mimics leukemias with a BCR-ABL1 translocation, such as Chronic Myelogenous Leukemia (CML) and Acute Lymphoblastic Leukemia (ALL). Inhibition of the oncogenic Abl kinase with imatinib reverses transformation, allowing progression to the next stage of B cell development. We employed a genome-wide polysome profiling assay called Gradient Encoding to investigate the extent and potential contribution of translational regulation to transformation and differentiation in v-Abl-transformed pre-B cells. Over half of the significantly translationally regulated genes did not change significantly at the level of mRNA abundance, revealing biology that might have been missed by measuring changes in transcript abundance alone. We found extensive, gene-specific changes in translation affecting genes with known roles in B cell signaling and differentiation, cancerous transformation, and cytoskeletal reorganization potentially affecting adhesion. These results highlight a major role for gene-specific translational regulation in remodeling the gene expression program in differentiation and malignant transformation.

## Introduction

B cells are the antibody-producing lymphocytes of the immune system. *Ex-vivo* transduction of mouse bone marrow with the viral oncogene v-Abl, a constitutively active version of the cellular c-Abl protein tyrosine kinase, suspends B cell differentiation in a permanently proliferating state (at the pre-B cell stage) [1], [2], [3]. This transformed state is a model for Chronic Myelogenous Leukemia (CML) and Acute Lymphoblastic Leukemia (ALL), of which 95% and 30% of cases, respectively, harbor the BCR-ABL1 translocation (referred to as Ph^+^) that leads to a constitutively active Abl kinase [4], [5], [6]. Primary Ph^+^ hematopoietic progenitors display reduced adhesion to the extracellular matrix, and stimulating adhesion of these cells in culture reduces proliferation, suggesting that their rampant proliferation is at least in part due to the inability to activate adhesion molecules [7], [8], [9], [10]. The kinase activity of both Abl and its oncogenic fusion derivatives can be inhibited by the small molecule drug, imatinib, which has dramatically improved prognosis for patients with leukemias harboring the BCR-ABL1 translocation [11], [12]. Unfortunately, many initially sensitive cancers develop resistance to imatinib, emphasizing the need for a more complete understanding of the molecular mechanisms in BCR-ABL1 transformation [13].

Cells transformed with v-Abl are arrested at the pre-B cell stage, as are leukemic cells in Ph^+^ ALL patients [14], [15]. Upon treatment with imatinib, v-Abl-transformed cells revert to a quiescent state, arresting in G1, and eventually undergo apoptosis, as do primary pro-B cells cultured in the absence of cytokines. RAG (Recombination Activating Gene) genes and transcription factors that activate Immunoglobulin (Ig) Light Chain locus rearrangements are induced, mimicking the transition from large, cycling pre-B I cells into small, resting pre-B II cells [16]. Several tumor-suppressor genes are also induced by imatinib treatment (and by inference repressed by v-Abl signaling), including Ku80, BRCA1, and Rb. Conversely myc, N-myc, and Lyl1 transcripts decrease in response to imatinib (and by inference are induced by v-Abl) [16]. Thus, this *ex vivo* system allows the identification of putative regulators and effectors of pre-B cell differentiation and/or transformation.

The transcriptional program induced by constitutive Abl kinase transformation is well studied [16], [17], [18], [19], [20] but the gene-specific translational program of Abl-transformed cells has not been thoroughly investigated. Translation is extensively altered in many cancers, including v-Abl transformed pre-B cells [21], [22]. The constitutively active Abl activates translation through the MAPK and PI3K/AKT pathways and leads to increased translation initiation via mTOR phosphorylation of RpS6 and 4E-BP1 [22]. Translation inhibitors have shown promise in battling Ph^+^ cancers [23] and the mTOR inhibitor rapamycin works synergistically with imatinib to stop proliferation of CML cell lines *in vitro* [24]. While the regulation of translation appears to contribute to the oncogenic transformation of v-Abl-transduced pre-B cells and some key proteins have been identified as post-transcriptionally regulated (namely c-Myc, MDM2, CEBPα, hnRNP E2/Pcbp2 and Cyclin D3) [25], [26], [27], [28], an unbiased, genome-wide survey of translational regulation in this cancer model has not been performed. Additionally, as mTOR inhibitors are gaining traction as therapeutics, it will be important to delineate the genes and pathways that v-Abl regulates independently of mTOR.

We analyzed the translational regulation of genes in wildtype Abl-transformed pre-B cells before and after treatment with imatinib (a v-Abl inhibitor) or rapamycin (an mTOR inhibitor). We used a streamlined version of traditional polysome profiling on a genomic scale where mRNAs within sequential fractions of a linear sucrose gradient were differentially labeled and analyzed by DNA microarray. This procedure, called Gradient Encoding, provides an accurate and reproducible ranking of the positions of mRNAs in the gradient, allowing sensitive detection of changes in the average number of ribosomes per mRNA, from which we infer the relative changes in translation rate of each mRNA from one condition to another [29].

## Results

### Gradient Encoding Results Are Concordant with Traditional Polysome Profiling

**Figure 1:**
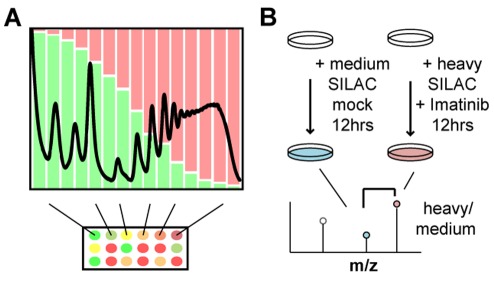
Schematics for the genomic (gradient encoding) and proteomic (pSILAC) techniques employed. A) Gradient encoding relies on separation of mRNAs in a sucrose gradient by virtue of the number of tethered ribosomes. mRNAs from each fraction are sequentially labeled with varying red/green ratio and analyzed by microarray. Each mRNA’s ratio of red/green represents its average position within the gradient. B) pSILAC involves metabolic labeling of proteins with either ^13^C-L-arginine (medium) or ^13^C-^15^N-L-Arginine (heavy) in place of ^12^C-^14^N-L-arginine only for the duration of drug- or mock- treatment (in pink or light blue, respectively) such that only proteins produced during treatment are labeled. The relative protein production is calculated by quantifying the ratio of treated to untreated (ie. heavy- to medium-labeled) peptides.

We used a genome-wide polysome profiling technique, called Gradient Encoding, to identify genes translationally regulated by v-Abl transformation, and to define the contribution of mTOR to this regulation. Lysates harvested before and after 12 hours of treatment either with 2.5 µM imatinib, a v-Abl kinase inhibitor, or 10 ng/mL (10.9 nM) rapamycin, an mTOR inhibitor, were fractionated by sedimentation through linear sucrose gradients. Gradient fractions were “encoded” such that the mRNA from successive fractions was labeled with increasing ratios of Cy5 to Cy3. mRNAs derived from fractions in the lighter portion of the gradient therefore have a lower Cy5 to Cy3 ratio, whereas those deeper in the gradient have a higher Cy5 to Cy3 ratio. The ratio of Cy5 to Cy3 for each mRNA therefore reflects its average position within the gradient (Fig. 1A). We thus “encoded” the sedimentation rate of each mRNA across the entire gradient. The resulting ratios were quantitatively measured for each mRNA species by hybridization to DNA microarrays, and related to the 260 nm absorbance peaks representing different numbers of ribosomes bound per mRNA (Fig. 1A, see Materials and Methods). We assume that changes in the number of ribosomes bound to a particular transcript in response to treatment represent a change in the rate of translation of that transcript, provided that 1) the sedimentation rate is determined by the number of ribosomes bound to each transcript, 2) initiation is rate-limiting and the elongation rate is uniform, and 3) the ORF length remains constant.

**Figure 2:**
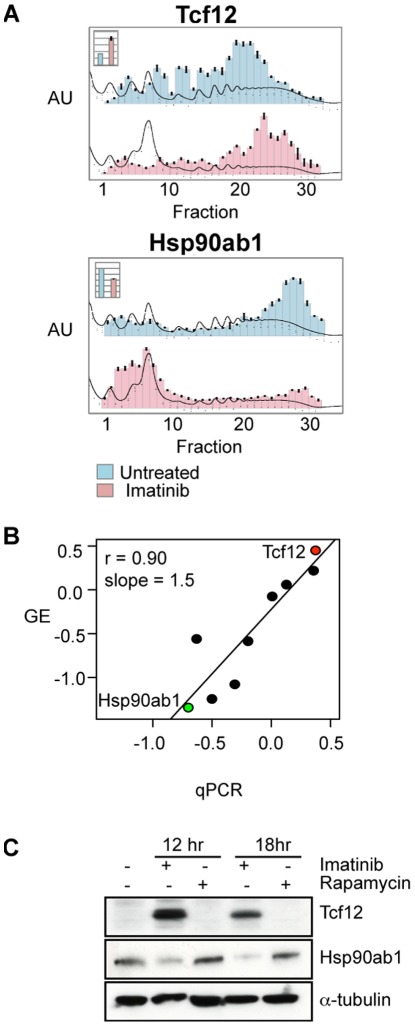
Changes in translation inferred by gradient encoding agree with those obtained by qRT-PCR of sucrose gradient fractions and Western analysis. A) qRT-PCR was performed on an equal portion of RNA harvested from each fraction from the high-resolution sucrose gradients performed on lysates from cells either un-treated (in light blue) or treated with imatinib (in pink). The histogram of relative mRNA abundance was superimposed over the A260 gradient trace. The y-axis represents relative absorbance in arbitrary units (AU) across each gradient, or relative mRNA abundance in each fraction. Inset) The relative ratio of mRNA abundance of Tcf12 or Hsp90ab1, as indicated, in the untreated (light blue) or imatinib-treated (pink) cytoplasmic lysates. (B) Comparison of estimated translation from high-resolution sucrose gradient fractionation followed by qRT-PCR on the x-axis (qPCR) to gradient encoding on the y-axis (GE) for 9 genes. Estimated translation for genes before and after imatinib treatment was explicitly calculated from the qRT-PCR experiments described in the Materials and Methods, and expressed as Log_2_ (treated/untreated). Values for genes represented by multiple oligos for Gradient Encoding were averaged. The Pearson correlation (r  = 0.90) and slope of the trend line (1.5) are shown. The points representing Tcf12 and Hsp90ab1 are colored red and green, respectively. C) Western analysis for Tcf12 and Hsp90ab1, with α-tubulin as a control, was performed on total lysates after 12 or 18 hours of the specified treatment.

We confirmed the Gradient Encoding results for nine selected genes by high resolution polysome profiling and RT-PCR before and after imatinib treatment (Fig. 2A, Fig. S1). The analysis for the two most extreme of these nine genes, Tcf12 (NM_011544) and Hsp90ab1 (NM_008302), is shown here (Fig. 2A). The relative change in mRNA abundance was measured simultaneously by quantitative RT-PCR (qRT-PCR) and is displayed in the insets of the gradient profiles (Fig. 2A, inset).

We quantitatively analyzed changes in transcript abundance and translation following imatinib or rapamycin treatment in three biological replicate experiments; all three replicate experiments were used in subsequent statistical analyses. The change in average ribosome number between conditions is expressed as the Log_2_ of the ratio of average ribsome number in treated versus untreated cells for each mRNA. The transcripts with the largest negative values are therefore those with the greatest apparent reduction in translation upon imatinib or rapamycin treatment. To quantitatively evaluate the performance of the encoding method, we calculated the expected “encoded” values, based on the results from qRT-PCR analysis of individual fractions. The results from the individual gene qRT-PCR (qPCR) were in agreement with the results from Gradient Encoding (GE) (Pearson r = 0.90, Fig. 2B). Western analysis of Hsp90ab1 and Tcf12, the two most extreme examples of translational regulation confirmed by qRT-PCR, highlighted in green and red, respectively (Fig. 2B), confirmed the dramatic induction of Tcf12 after 12 hours of imatinib treatment and showed modest reduction of Hsp90ab1 after 12 and 18 hours of imatinib treatment (Fig. 2C). However, Hsp90ab1 protein levels were not measurably reduced after 12 or 18 hours of rapamcyin treatment, despite significant reduction in translation after 12 hours (the average Log2 ratio was −1.34 for imatinib and −0.86 for rapamycin). This may reflect a difference in protein stability, which is not measured by our genomic methods, a point which will be addressed below.

### Translational Regulation Contributes Significantly to Global Reprogramming of Protein Composition in v-Abl Transformation

**Figure 3:**
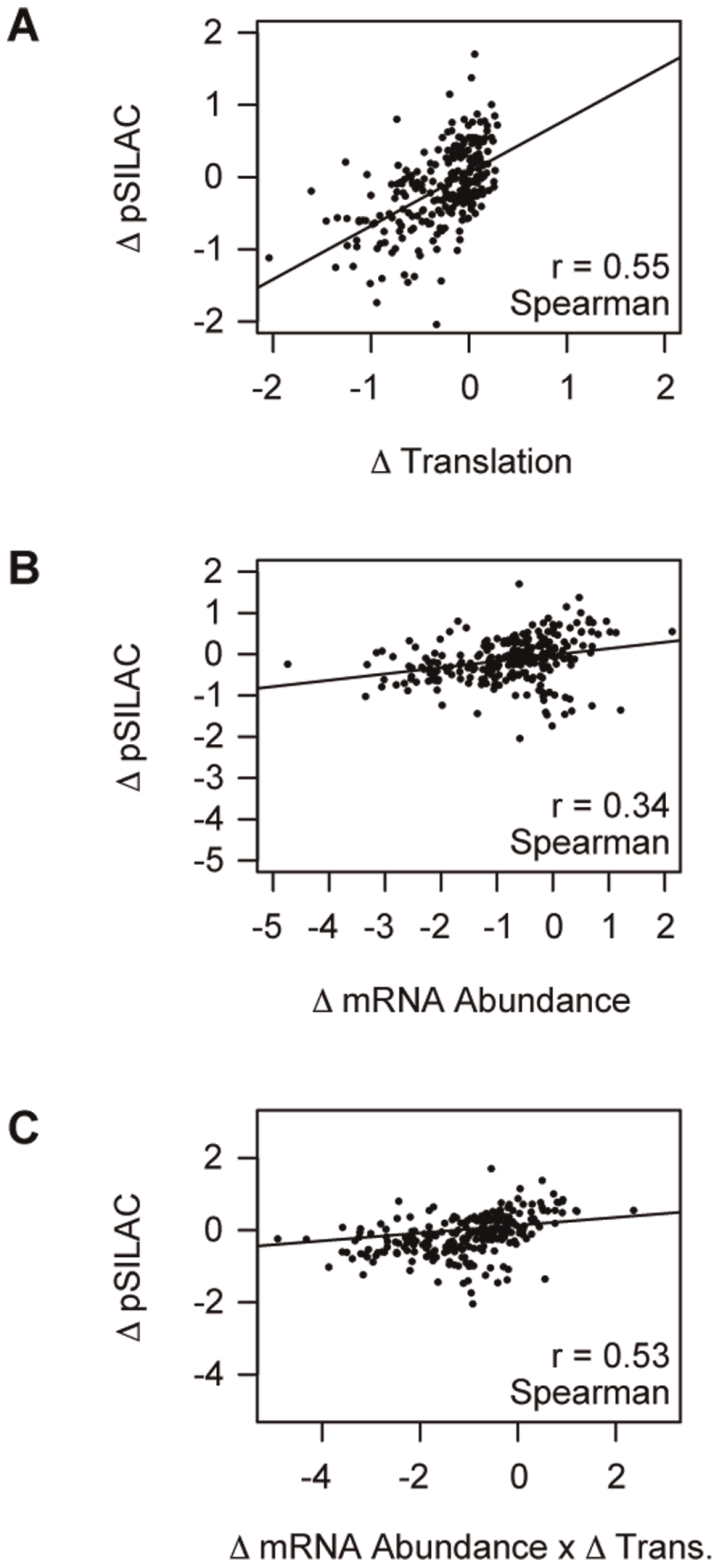
Changes in translation (inferred from gradient encoding) predict changes in protein abundance. A) Change in Translation versus Change in pSILAC. A scatter plot of the change in protein abundance as measured by pSILAC vs. the change in translation by Gradient Encoding, each expressed as the Log_2_ (treated/untreated). Trend lines and r values for the Spearman correlations between the two datasets are shown in black. B) Change in mRNA Abundance versus Change in pSILAC. As in “A”, but the x-axis is the change in mRNA abundance. C) Product of the Change in the mRNA Abundance and Change in Translation versus Change in pSILAC. As in “A”, but the x-axis is the Log_2_ (treated/untreated translation x treated/untreated mRNA abundance). Abund.  =  Abundance, Trans.  =  Translation.

We expected a change in translation to result in a corresponding change in protein production. Proteins with low turnover rates, however, might show only small changes in overall abundance even if their synthesis rates changed abruptly after 12 hours of drug treatment, as observed for Hsp90ab1 after rapamycin treatment (Fig. 2C). In order to more accurately measure gene-specific changes in protein synthesis over the course of drug treatment we used a pSILAC (pulsed Stable Isotopic Labeling by Amino acids in Cell culture) method whereby proteins are metabolically labeled only for the duration of drug treatment (Fig. 1B) [30]. v-Abl-transformed cells were cultured in standard “light” media and then switched to either “medium” or “heavy” media containing L-arginine with stable isotope-labeled carbon (^13^C-L-arginine) or both carbon and nitrogen (^13^C-^15^N-L-arginine), respectively, in place of ^12^C-^14^N-L-arginine upon addition of drug. Protein samples isolated from heavy-labeled, untreated cells and medium-labeled, imatinib-treated cells were mixed and ∼60 µg of this 1∶1 mixture of total lysate was subjected to PAGE (Polyacrylamide Gel Electrophoresis). The “reverse” experiment was performed comparing heavy-labeled, imatinib-treated cells and medium-labeled, untreated cells and served as a biological replicate. Each of the two lanes was cut into 8 slices that were separately analyzed by mass spectrometry. For each tryptic peptide that we could confidently identify, we determined the ratio of the medium to heavy isotope-labeled species, which should reflect the change in production of the corresponding protein after 12 hours of imatinib treatment. The data from both the “forward” and “reverse” experiments were used to determine the average ratios of imatinib-treated/untreated protein levels. We restricted this analysis to proteins for which at least two unique tryptic peptides were identified. To evaluate the relative contributions of translational and transcriptional regulation to the observed changes in protein production, we compared the correlation between changes in protein production +/− imatinib and the corresponding changes in estimated translation (Fig. 3A), transcript abundance (Fig. 3B), or the product of these two estimates (Fig. 3C) for 258 genes (see Table S1).

The observed changes in translation (Spearman r = 0.55, Pearson r = 0.55) more accurately predicted changes in protein production than did changes in mRNA abundance (Spearman r = 0.34, Pearson r = 0.27), suggesting that translation independently accounts for a large component of the reprogramming of the cell’s proteome (Fig. 3A-C). We include both Pearson (based on values) and Spearman (based on rank) correlations here, but only Spearman correlations are displayed in the figures. The genes for which we have pSILAC data qualitatively represent the total population, and are not skewed or clustered into regions of extreme change in either translation or abundance (Fig. S2).

The genes that made the greatest contribution to the better correlation of translation than transcription with protein synthesis rates were, as expected, components of the translation apparatus. Nevertheless, even when the ribosomal proteins, elongation factors, and initiation factors were excluded from the pSILAC dataset (resulting in the removal of data for 25 genes), the correlations between the changes in synthesis of specific proteins and either translation (Spearman r = 0.47, Pearson r = 0.48), abundance of the corresponding transcripts (Spearman r = 0.49, Pearson r = 0.44), or the product of change in mRNA abundance and translation (Spearman r = 0.60, Pearson r = 0.53) confirm the large contribution of translational regulation to reprogramming the protein composition of the cell.

Although gene-specific changes in protein turnover rates, which cannot be inferred from transcript abundance or translation rates, could play an important role, genome-wide quantitation of both changes in translation and changes in mRNA abundance greatly improves our ability to infer the global reprogramming of the cells proteome by enabling quantitative analysis of more than ten times as many genes as can be evaluated by current proteomic methods.

### A Major Role for mTOR in Reprogramming of Gene Expression by v-Abl

**Figure 4:**
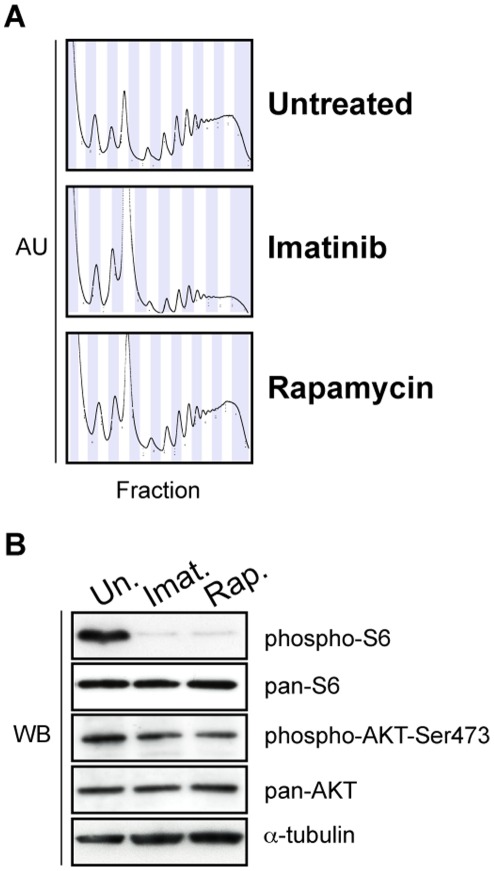
The translational response of v-Abl-transformed pre-B cells to imatinib (2.5 µM for 12 hours) or rapamycin treatment (10.9 nM for 12 hours) or no treatment. A) OD260 traces (arbitrary units, AU) of typical sucrose gradients (top of gradient is on the left) either untreated (Untreated), treated with 2.5 µM imatinib for 12 hours (Imatinib), or with 10 ng/mL rapamycin for 12 hours (Rapamycin). Alternating blue and white columns represent fractions. B) The same cytoplasmic lysates treated with imatinib (Imat.), rapamycin (Rap.), or untreated (Un.) that were used for gradient encoding were subjected to Western analysis using the antibodies indicated. Results are representative of three replicate experiments.

Concerted, large-scale activation of translation is a hallmark of the mTOR pathway [31], [32]. BCR-ABL1 induces phosphorylation of 4E-BP1 and S6K via mTOR through activation of both the PI3K and MAPK pathways [24]. Because the genes encoding components of the ribosome and translation complex subunits comprise a substantial fraction of the ribosome-associated mRNAs in dividing cells, the decreased translation of these genes may explain much of the global redistribution of mRNAs from polysomes to monosomes, reflected in the 260 nm trace of the polysome profile following imatinib treatment (Fig. 4A, and [22]).

Qualitatively, rapamycin treatment produced a less prominent shift in ribosomes towards monosomes than did imatinib (Fig. 4A). Rapamycin sterically hinders formation of mTORC1, but not mTORC2 [33] and mTORC1 inactivation can activate mTORC2, due to loss of a negative feedback from mTORC1 on the mTORC2 complex [34]. However, chronic exposure to rapamycin has been shown to also inhibit mTORC2 [35]. We tested the degree of mTORC1 and mTORC2 inhibition by the loss of phosphorylation of Ribosomal protein S6 (RpS6) (an mTORC1 target) or AKT-Ser473 (an mTORC2 target) (Fig. 4B). No augmentation of mTORC2 activation by rapamycin was observed; phosphorylation of RpS6 and AKT-Ser473 was reduced to a comparable extent by either imatinib or rapamycin, at the concentrations and duration of drug used here.

**Figure 5:**
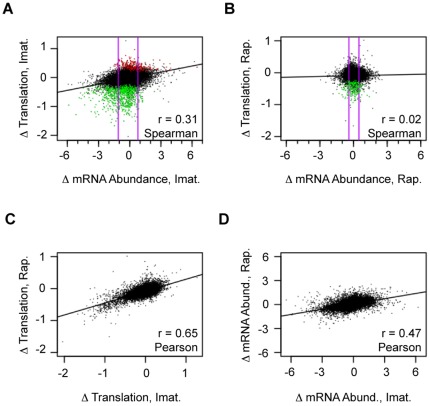
Changes induced by imatinib correlate with changes induced by rapamycin. A) Imatinib Change in mRNA Abundance versus Change in Translation. Red points represent the genes that were deemed statistically significantly translationally repressed by v-Abl (and activated by imatinib) (FDR <10%, SAM). In green are those statistically significantly translationally activated by v-Abl (and repressed by imatinib) (FDR <10%). Purple vertical lines define one standard deviation from the mean change in mRNA abundance and the trendline is shown in black. Spearman correlations are given. B) The Rapamycin Change in Abundance versus Change in Translation. As in A) but for rapamycin. C) Change in Translation for Imatinib versus Rapamycin. The trendline is in black and Pearson correlation is given. D) Change in mRNA Abundance for Imatinib versus Rapamycin. The Pearson correlation is given. All values are expressed as Log_2_ (treated/untreated). Imat.  =  imatinib, Rap.  =  rapamycin, Abund.  =  abundance.

mTOR canonically induces translation of genes harboring a 5′TOP sequence [36]. The requirement of this signal for mTOR-dependent translational regulation, however, is not stringent, and cell-type-dependent responses to mTOR inhibition are common [37]. Therefore, we compared the translational reprogramming after imatinib to that after rapamycin treatment in order to determine the contribution of mTOR to the translational re-programming of v-Abl. Figure 5A shows genes statistically significantly activated (in red) by imatinib (by inference repressed by v-Abl) and repressed (in green) by imatinib (by inference activated by v-Abl) in scatterplots relating changes in translation to mRNA abundance (Fig. 5A). Of the 10507 unique genes for which we had data in three replicate experiments, 175 unique genes were deemed significantly activated by imatinib (10% FDR (false discovery rate) cut off, using significance analysis of microarrays (SAM)), and 591 genes were repressed (10% FDR cut off, SAM). Despite equal dephosphorylation of RpS6 and AKT-Ser473, the range of change in translation affected by rapamycin was smaller than that following imatinib treatment. None of the 10688 unique genes for which we acquired rapamycin data in triplicate were translationally induced and 192 unique genes were repressed at a 10% FDR threshold (Fig. 5B).

Despite the difference in magnitude, the pattern of changes in translation after imatinib treatment correlated well with changes following rapamycin treatment (Pearson r = 0.65, Fig. 5C), suggesting that mTOR activation could in principle account for much of v-Abl induced translational induction. In addition, rapamycin-induced changes in mRNA abundance correlated quite well with those induced by imatinib (Pearson r = 0.47, Fig. 5D) suggesting that mTOR activation could also (perhaps indirectly) account for a substantial component of the change in mRNA abundance in these cells. However, there are isolated cases where imatinib and rapamycin caused discordant effects on the translation of specific genes, which will be discussed below.

### Extensive Gene-Specific Reprogramming of Translation Following Abl Inhibition

Imatinib treatment induced gene-specific changes in mRNA abundance that generally paralleled the corresponding changes in translation (Spearman r = 0.31, Fig. 5A). Despite this overall correlation, 109 of the 175 genes with significant increases in translation and 390 of the 591 genes with significantly reduced translation showed changes in mRNA abundance that were within one standard deviation (SD) of the mean for all detectable mRNAs (Fig. 5A, purple lines represent 1 SD). Translational regulation thus appeared to be the principal mode of regulation for more than half of the ∼770 genes either activated or repressed by v-Abl (see Tables S2 and S3, respectively).

**Figure 6:**
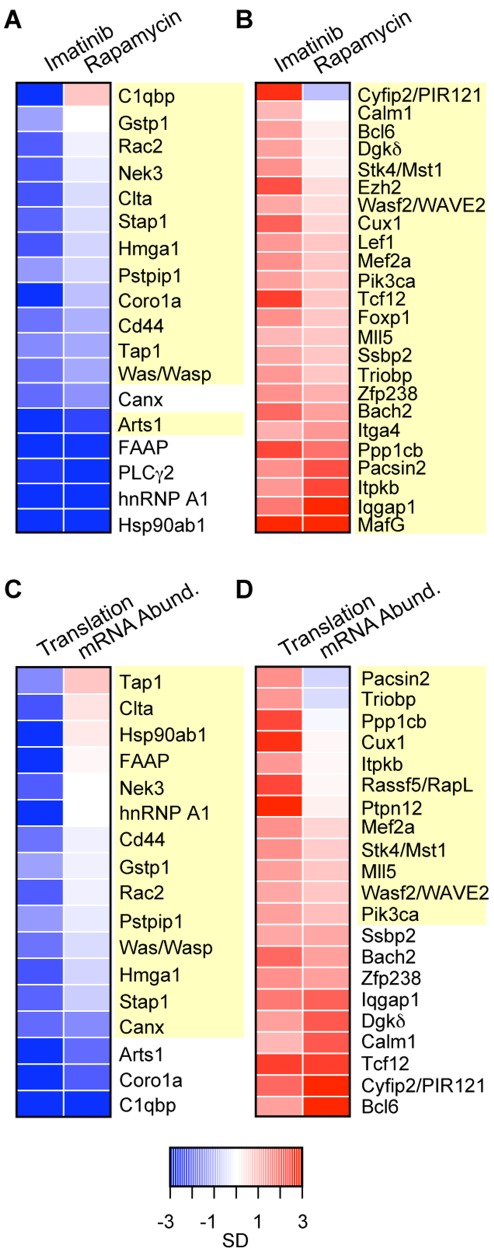
Heatmaps depicting changes in translation upon imatinib and rapamycin treatment for genes either significantly translationally activated (A,C) or repressed (B,D) by v-Abl. Normalized average change (median = 0, SD = 1) was used to display changes in translation upon treatment with imatinib versus rapamycin as indicated (A,B) or changes in translation versus changes in mRNA abundance upon treatment with imatinib (C,D). Heatmaps are ordered by increasing change in translation upon rapamycin treatment (A,B) or by increasing change in mRNA abundance upon imatinib treatment (C,D). In cases where multiple oligos represent a gene, the oligo with the biggest change in translation upon imatinib treatment was used. Genes that were regulated translationally by imatinib but not rapamycin (A,B) or regulated translationally but not at the level of mRNA abundance by imatinib treatment (C,D) are highlighted in yellow (10% FDR cutoff, SAM). (SD, standard deviation).

Genes with functional roles in translation were highly over-represented (p<10^−32^, GO term enrichment) among the genes whose ribosome density decreased upon addition of imatinib, very likely reflecting the activation of mTOR by v-Abl. Genes encoding mitochondrial proteins (p<10^−11^, GO term enrichment) were also over represented in this group, suggesting that v-Abl activity can promote translation of genes involved in aerobic respiration. In general, the translational response to rapamycin was similar but weaker than that to imatinib for both mitochondrial and translation apparatus genes (Tables S3 and S4). Comparing average changes in translation across experiments confirms the general concordance between rapamycin and imatinib translational regulation for these subsets with one notable exception; C1qbp (Complement 1q-binding protein) is translationally induced by imatinib treatment while rapamycin has the opposite effect (Fig. 6A, Fig. S3A). C1qbp is a mitochondrial/cell surface protein implicated in promoting tumorigenesis [38], [39].

Genes involved in antigen processing and presentation were also significantly over-represented among the transcripts whose translation was inhibited by imatinib (p<0.025, KEGG pathways, Tables S2 and S4). A similar pattern in response to rapamycin treatment (Fig. S3A) suggests that the inhibition of MHCI antigen presentation canonically seen upon rapamycin treatment is due not only to reduced translation in general, but also to specific translational regulation of proteins participating in the antigen presentation pathway, such as Tap1, Erap1/Arts and constituents of the proteasome.

Three genes activated by v-Abl caught our attention due to their involvement in leukemia and cancer: Hmga1 (NM_016660), hnRNP A1 (NM_010447), and Hsp90ab1. Both hnRNP A1 and Hsp90ab1 have previously been reported to play important roles in BCR-ABL1-mediated leukemogenesis [40], [41], [42]. Although HMGA1 is a known oncogene, to our knowledge, it has not previously been implicated in v-Abl–mediated transformation. All three of these genes displayed almost complete translational arrest upon imatinib treatment (Fig. 2A, Fig. S1). The abundance of these mRNAs also decreased, but only by roughly 1.5 to 2 fold (Fig. 2A, Fig. S1, Fig. S3B). These data suggest that v-Abl activates the expression of these genes predominantly by promoting their translation. Translation of hnRNP A1 and Hsp90ab1 was also significantly translationally repressed by rapamycin treatment, but the change in Hmga1 translation upon rapamycin treatment was not significant by SAM (local FDR 88%, Fig. 6A, Fig. S3A), and was less than one standard deviation from the mean, suggesting that mTOR activation is responsible at least in part for the v-Abl dependent translational induction of Hsp90ab1 and hnRNP A1, but not significantly for translational induction of Hmga1 (Fig. 6A). The abundance of all three transcripts changed only slightly in response to treatment (Fig. 6C, Table S3B, Fig. S1).

Although the predominant effect of v-Abl inhibition was to decrease translation, ∼160 genes were translationally activated upon imatinib treatment with a striking enrichment for genes encoding nuclear proteins (p<10^−11^, GO term enrichment) and proteins involved in cytoskeletal reorganization, particularly integrin activation.

Nuclear proteins translationally repressed by v-Abl included transcription factors implicated in repressing the IgH locus (Ezh2 (NM_007971), Cux1/Cutl1 (NM_009986), Bach2 (NM_007521), perhaps with MafG (NM_010756)) [43], [44], [45], [46], [47], activating RAG and/or promoting B cell differentiation (Foxp1 (NM_053202), Lef1 (NM_010703), Bcl6 (NM_009744)) [48], [49], [50], [51], and tumor suppression (Ssbp2 (NM_024186), Mll5 (NM_026984)) [52], [53]. Others had no previously identified role in B cell development but could putatively enhance κlocus activation due to their ability to interfere with Id protein inhibition of E2A products (Mef2a (NM_013597), Tcf12 (NM_011544), Zfp238 (NM_013915)) (see Table S2 for a complete list).

**Figure 7:**
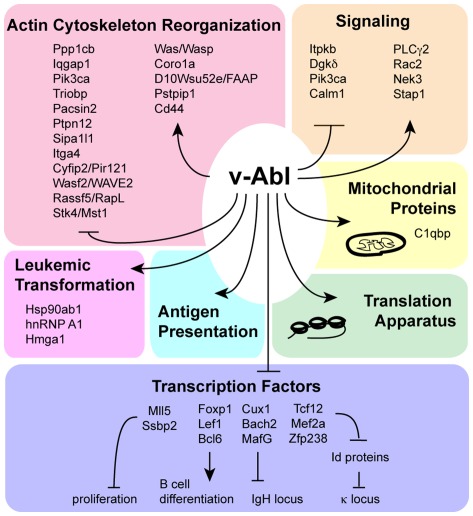
A summary of the contribution of v-Abl translational re-programming to differentiation arrest and leukemogensis.

Discordant yet significant regulation of multiple genes with shared functional roles was observed for genes involved in actin cytoskeleton reorganization and the phosphoinositide 3-kinase (PI3K) pathway, both of which were enriched in the v-Abl-repressed gene set (p<0.037, KEGG pathways, Table S4). The PI3K pathway (induced downstream of the pre-BCR) was over-represented in the v-Abl-repressed gene set; they included Itpkb (Inositol 1,4,5-trisphosphate 3-kinase B) (NM_001081175), Dgk δ (DAG kinase δ) (NM_177646), Pik3ca (PI3K catalytic subunit 110α) (NM_008839), and Calm1 (Calmodulin 1) (NM_009790), although a number of molecules in convergent pathways were translationally induced by v-Abl, including PLCγ2 (NM_172285), Rac2 (NM_009008), Nek3 (NM_011848), and AI586015/Stap1/BRDG1 (BCR Downstream Signaling 1) (NM_019992) [54], [55], [56]. The set of genes repressed by v-Abl was enriched for involvment in actin cytoskeleton reorganization, represented by Ppp1cb (NM_172707), Cyfip2/Pir121 (NM_133769), Iqgap1 (NM_016721), Itga4 (NM_010576), Pik3ca (NM_008839), Wasf2/WAVE2 (NM_153423), Rassf5/RapL (NM_018750). Additional v-Abl-repressed genes potentially involved in cytoskeletal reorganization but not included in the KEGG annotations include Stk4/Mst1 (NM_021420) [57], Ptpn12 (NM_011203) [58], Pacsin2 (NM_011862) [59], Triobp (NM_138579) [60], and Sipa1l1 (NM_172579) [61] (Fig. 7). A few regulators of actin reorganization, Was/Wasp (NM_009515) [57], Coro1A (Coronin-1) (NM_009898) [57], D10Wsu52e/FAAP (NM_145422) [62], and Pstpip1 (NM_011193) [58], were translationally induced by v-Abl.

The significant repression of Cyfip2/Pir121, Wasf2/WAVE2, Rassf5/RapL, Stk4/Mst1 and Itga4 by v-Abl is notable due to their concordant involvement in TCR-mediated integrin activation [57]. Conversely translation of another molecule important for hematopoietic cell adhesion, Cd44 (NM_009851), is induced by v-Abl [63]. The discordant regulation of functionally related genes demonstrates the gene-specific nature of v-Abl’s translational regulation, and suggests its involvement in re-wiring pathways affecting BCR signaling and adhesion.

In contrast to genes encoding mitochondrial proteins and the translation apparatus, those involved in cytoskeletal reorganization, BCR signaling, and transcription factors had more varied responses to rapamycin (Fig. 6A-B and Fig. S3A). None of the genes translationally activated by imatinib treatment were significantly induced by rapamycin treatment at the 10% FDR cutoff. The gene whose translation was most differentially regulated in response to imatinib was Cyfip2, a gene whose overexpression single-handedly increases TCR-mediated adhesion [64](Fig. 6B).

Using the same significance criteria (10% FDR, SAM), more than twice as many genes showed significant changes at the level of mRNA abundance than at the level of translation, yet there is limited overlap between these two groups. Fewer than half of the translationally regulated genes distinguished by GO term or KEGG pathway analysis discussed above also significantly changed at the level of mRNA abundance, stressing the large amount of information missed by only considering changes in mRNA abundance (Fig. 6C-D, Fig. S3B). Over half of the transcription factors with significant translational changes did not significantly change at the level of mRNA abundance. Moreover, GO term and KEGG pathway analysis failed to identify antigen processing, lysosome constituents, the phosphatidylinositol signaling system or regulation of actin cytoskeleton as enriched categories using genes significantly changing only at the level of mRNA abundance (using either the same number of genes as used for translation, or the same 10% FDR cutoff, SAM). A table of all combined data is provided (Data S1).

v-Abl orchestrates a dynamic, gene-specific program of post-transcriptional regulation of the basic cell machinery necessary for rapid proliferation, increasing expression of oncogenes, as well as re-wiring signaling pathways that govern cell fate decisions (Fig. 7). The selective translational activation or repression of diverse genes in response to imatinib treatment suggests that extensive reprogramming of translation is likely to play an important part in the ability of activated Abl to arrest B cell differentiation and promote leukemic transformation.

## Discussion

### An Independent Role for Translational Reprogramming

We found that translational regulation plays a major role in v-Abl-mediated reprogramming of the proteome during B cell differentiation and transformation. We observed significant translational activation or repression of almost 800 genes in response to imatinib treatment. For more than half of these the corresponding change in mRNA abundance was less than 1SD from the mean, suggesting that translation plays an important, independent role in v-Abl-mediated regulation of gene expression in this system.

We compared the observed changes in translation to changes in protein production using pSILAC (Fig. 1B) [30], where newly synthesized proteins are metabolically labeled only for the course of drug (or mock) treatment. We found extensive imatinib-sensitive reprogramming of translation that correlated with changes in protein production following imatinib treatment, including many cases where changes in mRNA abundance did not, suggesting that for many genes, the dominant mode by which gene expression is reprogrammed in response to Abl-activation is post-transcriptional. While examples of translation regulation in the absense of a shift of the corresponding mRNA in the gradient, or stalled complexes, exist [65], [66], the good correlation between changes in protein production (measured by pSILAC) and the changes predicted from changes in translation rates lends credence to our inference of translation rates from the sedimentation of mRNAs through the gradient. While our method does not provide specific information about ribosome position on the mRNA, an advantage of the recently-reported footprinting method [67], Gradient Encoding is less experimentally challenging and less expensive, and it should provide better quantification of less abundant mRNAs, such as those encoding transcription factors, for which the sparse coverage and biased sampling of sequence-based methods might be limiting. The ability to infer changes in the proteome from purely genomic data greatly increases the number of proteins whose regulation can be quantitatively evaluated including proteins that are currently difficult or impossible to measure directly by mass spectrometry or immunoassays.

A handful of genes have been previously shown to be post-transcriptionally regulated by BCR-ABL1. In agreement with previous results c-Myc was induced by v-Abl both transcriptionally and translationally, [16], [25]. Also in agreement with previous observations, hnRNP E2/Pcpb2 was significantly translationally repressed by imatinib, but the target of its post-transcriptional regulation, C/EBPα, is not expressed in this pre-B cell line [28]. Discrepancies between our data and previous reports of translational effects of BCR-ABL1 in myeloid cells, such as translational (and transcriptional) induction of Cyclin D2, rather than D3 [27], and the absence of v-Abl-mediated translational induction of MDM2 in our murine cell line (where we observed modest v-Abl-mediated repression of MDM2 translation) [26], are likely to reflect cell-type specific features of this translational program. Indeed, genes downstream of mTOR translational regulation are known to be cell-type specific [37] most likely due to the presence of cell-type specific trans-acting factors mediating mTOR-induced translational regulation.

### mTOR Activation Explains Much but not all of v-Abl-mediated Translational Reprogramming

mTOR inhibitors have gained attention for their efficacy in combating Ph+ leukemias [23]. Understanding mTOR-dependent and -independent aspects of v-Abl-mediated transformation should indicate pathways not effectively targeted by mTOR inhibitors. We were able to assess the contribution of the mTOR pathway to v-Abl regulation of translation by comparing imatinib- to rapamycin-treated v-Abl-transformed cells. Rapamycin and imatinib inhibited mTORC1 activation equally well as measured by RpS6 phosphorylation (Fig. 4B). Inhibition of mTOR by rapamycin may not always be qualitatively or quantitatively complete. For example inhibition of mTOR by rapamycin can relieve S6 Kinase-mediated inhibition of PI3K [34], a negative feedback mechanism in the mTOR pathway, which can lead to activation of mTORC2. Although in our experiments mTORC2 activation, as measured by phospho-Ser-473 AKT, was not increased by rapamycin treatment, we cannot exclude the possibility that other mechanisms make rapamycin less efficient than imatinib in inhibiting the mTOR pathway.

Although much of the v-Abl-mediated translational activation appears to be through mTOR, individual genes translationally regulated by v-Abl vary in their dependence on mTOR (Fig. 6A-B, Fig. S3A). mTOR appeared to be responsible for the increased translation of many components of the translation apparatus as well as a large number of mitochondrial proteins. In Jurkat cells, disruption of mTOR-raptor interactions critical for functional mTORC1 (the rapamycin-sensitive mTOR complex) have been correlated with a lower mitochondrial membrane potential [68]. BCR-ABL1-positive cells produce ROS (reactive oxygen species) due to PI3K/mTOR-mediated over-activation of electron transport [69]. Our results suggest the possibility that v-Abl/mTOR-mediated translational activation of the enzymes involved in mitochondrial ATP production contributes to these responses. The mitochondria-associated gene most differentially translationally regulated by imatinib and rapamycin, C1qbp (NM_007573) (imatinib significantly suppresses while rapamycin slightly increases its translation), has been implicated in suppressing mitochondrial-mediated cell death, thus preventing apoptosis despite ROS overproduction [39](Fig. 6A, Fig. S3A). Pathways involved in such “non-oncogene dependencies” are now recognized as possible cancer-specific therapeutic targets [70], [71]. Gstp1 (Glutathione-S-transferase pi 1), previously implicated to play such a role, is similarly translationally activated by v-Abl and significantly repressed by imatinib but not rapamycin treatment (FDR  = 85%) [70]. Thus, our data suggest that there is an rapamycin-insensitive component to v-Abl’s translational activation of these ROS-controlling genes. Similar cases of predominantly rapamycin-insensitive v-Abl regulation will be discussed below.

### Oncogenes are Translationally Induced by v-Abl

We identified three genes involved in leukemogenesis whose translation was markedly repressed by imatinib; Hmga1, hnRNP A1, and Hsp90ab1. HMGA1 is a known oncogene with no previously identified role in Abl signaling. hnRNP A1 influences cap-dependent translation [72] and plays roles in RNA splicing and nuclear export [73] and its nucleo-cytoplasmic shuttling activity may be necessary for BCR-ABL1-induced leukemogenesis; indeed, levels of this protein increase in patients in CML blast crisis (the terminal phase of the disease) [40]. Hsp90ab1 translation was repressed and protein levels were diminished following imatinib treatment (Fig. 2A, 2C). Inhibiting Hsp90ab1 with geldanamycin and 17-allylaminogeldanamycin (17-AAG) leads to BCR-ABL1 degradation and inhibited cell growth in BCR-ABL1 expressing cells [74]. Its repression in response to imatinib treatment (and by inference activation by v-Abl) suggests that it might participate in a positive feedback loop that maintains activation of this pathway. Translation of these three genes was also inhibited by rapamcyin treatment, although only modestly if at all for Hmga1 (FDR >87%, Fig. 6A), suggesting that they are regulated in part through the mTOR pathway.

### v-Abl Represses Translation of Differentiation-Promoting Transcription Factors

Transcription factors are highly enriched (p<0.0028, Genetrail, GO term enrichment, see Table S4 for a complete list) among the genes translationally-repressed by v-Abl; many of these have been previously implicated in regulating B cell development, including Foxp1, Lef1, Bcl6, Bach2, and Cux1. Foxp1 and Lef1 are required for proper RAG2 expression and B cell development [48], [75], [76]. Bcl6 regulates the pro- to pre-B cell transition by protecting pre-B cells undergoing Light Chain rearrangement from DNA damage-induced apoptosis and also prevents plasma cell differentiation in concert with Bach2 [50], [77], [78]. In addition, Bach2 represses a reporter construct containing the IgH enhancer in partnership with a small, as yet unidentified, Maf protein [47]. The coordinate translational activation of MafG suggests it as a prime candidate for this role. Cux1, which affects double positive (DP) T cell development by repressing the TCRβ enhancer, similarly binds regions throughout the IgH V (V_H_) genes and represses the IgH intronic enhancer *in vitro* [44], [45], [79]. The concomitant translational induction of proteins implicated in repressing the IgH locus (Cux1, Bach2, and possibly MafG) upon induction of pre-B cell differentiation could implicate their involvement in allelic exclusion.

Light Chain (ie. κ locus) activation is sensitive to the dosage of basic Helix-loop-helix (bHLH) E2A proteins [80] which are inhibited by binding to Id proteins [81], [82]. Translational induction of the bHLH proteins Mef2a and Tcf12 (whose protein is dramatically induced, Fig. 2C) might enable them to compete with the E2A proteins for binding to the inhibitory Id proteins, thus promoting κ locus activation. The overlapping and coordinated roles of these transcription factors in B cell development are mirrored in their synchronized translational activation upon imatinib treatment.

### v-Abl Regulates Translation of Molecules in pre-BCR Signaling Pathways

pre-B cells require signals from the pre-BCR in order to proliferate, and BCR-ABL1-positive pre-B cells bypass this requirement by mimicking some aspects of pre-BCR signaling [83]. However, the ability to signal downstream of the pre-BCR is selected against in Ph^+^ leukemias, and BCR-ABL1-mediated repression of pre-BCR-induced Ca^2+^ signaling has been described in Ph^+^ B-ALL patients, and in a mouse ALL model [14], [15], [83]. Additionally, v-Abl-transformed pre-B cells resemble pre-B cells that have not received the pre-BCR signal in that they continue to rearrange Heavy Chain loci in the presence of a successfully rearranged Heavy Chain, thus failing to induce allelic exclusion, and the Light Chain loci are inactive [16], [84]. Treatment of v-Abl transformed pre-B cells with imatinib has been previously shown to mimic pre-BCR signaling, with activation of the RAG proteins and subsequent Light Chain rearrangements [16]. Thus the constitutively active Abl mimicks the proliferative signals induced by the pre-BCR, but prevents the calcium flux and differentiation downstream of pre-BCR signaling. Our data show that a number of genes that influence these pathways are translationally regulated by v-Abl, some independent of changes in mRNA abundance and some with no measurable contribution from mTOR (Fig. 6).

PI3K signaling through a Tec kinase/Rac2/Vav pathway is responsible for the pre-BCR-induced calcium flux described above [85]. Our results show that v-Abl translationally represses constituents of the phosphatidylinositol signaling pathway (p<0.037, KEGG pathways), as evidenced by the imatinib-dependent translational activation of Pik3ca- the catalytic subunit of PI3K, Calm1, Itpkb, and Dgkδ. Conversely, a set of genes likely to positively regulate the phosphatidylinositol signaling pathway were translationally repressed upon imatinib treatment: PLCγ2, Rac2, AI586015/Stap1/BRDG1 (BCR Downstream Signaling 1) and Nek3 [54], [55], [56]. Nek3, while not directly implicated in pre-B cell signaling, phosphorylates Vav1 and 2 in a breast cancer line [55], [56]. Stap1 provides a positive feedback loop for Tec kinases downstream of BCR signaling [54]. The transforming ability of the constitutively active Abl kinase is dependent on activation of the PI3K pathway, Rac2 and Vav. [86], [87], [88], [89], [90], [91]. Indeed a dominant negative Rac prevents ERK activation and induction of proliferation by v-Abl [88], [89].

The signal from the pre-BCR normally promotes either differentiation or cell death [92]. The majority of the pre-B cells from the Ph^+^ ALL mouse model mentioned above lacked a functional pre-BCR, and restoration of the pre-BCR signal induced calcium flux and cell death [14], [15]. Perhaps this translational re-programming contributes to v-Abl’s ability to bypass the necessity of a pre-BCR signal for proliferation (by activating the PLCγ2/Rac2/Vav pathway), while preventing a concerted calcium flux that would either result in cell death or differentiation (by repressing constituents of the phosphatidylinositol pathway). Rapamycin caused changes in translation of Rac2, Stap1, and Nek3 that were not significant by SAM and no changes at the level of abundance were observed upon imatinib treatment (the lowest local FDR for either change in rapamycin-induced translation or imatinib-induced changes in transcript abundance being 78%).

### v-Abl Represses Translation of Adhesion-Activating Molecules

pre-BCR-mediated calcium flux induces adhesion via integrin activation [85]. The Abl protein interacts with a number of cytoskeletal proteins and is known to alter protein interactions at sites of adhesion [93]. Our data provide evidence that Abl influences cytoskeletal re-organization at the level of translational regulation as well. v-Abl translationally represses five key proteins involved in TCR-mediated integrin activation including Wasf2/WAVE2, Stk4/Mst1, Rassf5/RapL and Cyfip2, as well as a subunit of one of the integrins implicated in Ph^+^ CML adhesion, Itga4 [10], [57]. Another cytoskeletal protein translationally repressed by v-Abl, IQGAP1, both co-localizes at sites of adhesion in NK-like cells and co-immunoprecipitates with Rac2 and RACK1 (receptor for activated C kinase) and is thought to target calcium/calmodulin signaling to the cytoskeleton [94], [95], [96]. Recently mTOR signaling has been implicated in regulating the translation of factors important for metastasis in prostate cancer [97] suggesting that translational control of adhesion and migration may be a common theme in cancer pathogenesis.

Reduced adhesion to bone marrow stroma and early egress from the bone marrow are hallmarks of Ph+ CML hematopoietic progenitors; translational repression of integrin-activation molecules could contribute to this phenotype. However, reports of BCR-ABL1 expression increasing adhesion in the Ba/F3 pro-B cell line [98], [99] raise the alternative possibility that the apparent reduced translation observed when v-Abl is active could be a result of localized translation necessary for activation of these adhesion molecules, since localized translation inherently requires the repression of translation of the mRNAs while in transit, prior to proper localization.

Wasf2, Stk4, and Rassf5 did not change significantly at the level of mRNA abundance (lowest FDR being 27%). The change in translation of Cyfip2, Rassf5/RapL and Stk4/Mst1 upon rapamycin treatment was not significant by SAM (the lowest local FDR being 61%). In addition Cyfip2, which is alone sufficient for increased TCR-mediated adhesion, is induced by imatinib both transcriptionally and translationally yet repressed at both levels by rapamycin (Table S2) [57]. These data suggest that rapamycin and imatinib might have different effects on the adhesion properties of Ph^+^ cells.

### Conclusions

We have uncovered major changes in post-transcriptional regulation of genes with critical roles in transformation or differentiation, and identified additional genes with potential roles in these processes based on specific changes in their translational regulation. Gene-specific changes in net protein synthesis were better predicted by the measured changes in translation than by changes in mRNA abundance for ∼260 proteins quantitatively profiled by pulsed SILAC-mass spectroscopy. We propose that v-Abl-mediated gene-specific translational regulation contributes to the transformed phenotype and adhesion defects of Ph^+^ cells from CML patients, as well as the differentiation arrest of pre-B cells observed in v-Abl transformed bone marrow and Ph^+^ ALL. These results highlight the critical role of gene-specific translational regulation in shaping the proteomic landscape in both cancerous transformation and differentiation.

## Materials and Methods

### Cell Lines and Cell Culture

Abl-transformed cells (E2A^+/+^ [80]) were maintained in RPMI 1640 with Glutamine added (Gibco, cat.# 22400-121) supplemented with 5%, heat-inactivated FCS (HyClone), 1x Pen/Strep (Gibco cat.# 15140122) and 50 mM β-mercaptoethanol. Cells were incubated at 37°C with 5% CO_2_. The kinase inhibitor imatinib (imatinib mesylate, aka. STI-57I or Gleevec) (Novartis) was prepared as a 10 mM stock solution in EtOH, sterile filtered, and stored in aliquots at –20°C and used at a concentration of 2.5 µM. The mTOR inhibitor rapamycin was prepared as a 5 mg/mL stock in EtOH and used at a final concentration of 10 ng/mL. For SILAC analysis, v-Abl transformed pre-B cells were grown in light media as per the SILAC protein quantitation kit-RPMI 1640 (Pierce Thermo Scientific cat.# 89982). Cells were washed in SILAC media without L-arginine twice, and then resuspended in either media containing 50 mg/500 mL ^13^C-L-arginine (medium) or 50 mg/500 mL ^13^C-^15^N-L-Arginine (heavy) in place of ^12^C-^14^N-L-arginine and immediately either mock or imatinib-treated. Both medium and heavy media were supplemented with 115 mg/L proline to limit metabolic conversion of arginine to proline [100].

### Preparation of Cell Extracts for Translation Profiling

For translation experiments, ∼60–100 million v-Abl transformed pre-B cells, either treated or untreated with imatinib or rapamycin (see Cell Culture) were harvested by resuspension in ice-cold buffer A (20 mM Tris pH 8.0, 140 mM KCl, 5 mM MgCl_2_, 0.1 mg/ml cycloheximide (Calbiochem Cat.# 239764). After the second wash, cells were resuspended in lysis buffer which was buffer A containing 0.22 mg/ml of heparin, 1× protease inhibitor cocktail (Pierce Cat.# 78437), 100 U/ml SUPERASin (Ambion Cat.# AM2696), and 0.5 mM DTT, 0.1% Brij 58 (Sigma Aldrich Cat.# P5884-100G) and 0.1% sodium deoxycholate (Sigma Aldrich Cat.# D6750-100G). Cells were kept on ice for 15 min with intermittent inversion and then spun at 9,500 rpm in a microcentrifuge for 5 min at 4°C yielding ∼6–10 A260 units. Supernatant was collected, flash frozen in liquid nitrogen, and stored at −80°C until use.

### Western Analysis

For Tcf12 and Hsp90ab1 Westerns, equal numbers of cells were extracted with RIPA buffer (25 mM Tris-HCl pH 7.6, 150 mM NaCl, 1% NP-40, 1% sodium deoxycholate, 0.1% SDS), spun at maximum speed for 5 min., and an equal volume of supernatant was run on SDS-PAGE, transferred, and blotted with anti-Tcf12 (Protein Tech group cat.# 14419-1-AP) or anti-Hsp90beta (Stress Marq cat.# SMC-136), respectively. For anti-pan and anti-phospho- AKT or RpS6, equal A260 units of the same cytoplasmic cell extracts used for gradient encoding were subjected to PAGE, transferred, and blotted with anti-phospho RpS6 (Cell Signaling cat.# 4838), anti-pan RpS6 (Cell Signaling cat.# 2317), anti phospho-Ser473-AKT (Cell Signaling, cat.# 4058S), or anti-pan-AKT (Cell Signaling cat.# 9272). Anti-α tubulin (SIGMA, cat.# T6189) was used as a control for both assays. All antibodies were used as per vendor instructions.

### Sucrose Gradient Preparation

Sucrose gradients were prepared using the Gradient Master (Biocomp) according to the manufacturer’s suggestions. Five percent and 60% (w/v) sucrose solutions were prepared by dissolving sucrose in Gradient Buffer (20 mM Tris-HCl pH 8.0, 140 mM KCl, 5 mM MgCl_2_, 0.5 mM DTT, 0.1 mg/ml cycloheximide) at room temperature. The 60% solution was dispensed into an SW41 ultracentrifuge tube through a cannula underneath the 5% solution. Using an 11-step program (Biocomp, SW41 SHORT SUCR 5–50 11), the two solutions were mixed on the Gradient Master to form a linear gradient. After preparation, gradients were chilled for 30 min at 4°C.

### Sucrose Gradient Velocity Sedimentation

Immediately before centrifugation, ∼3.75 A260 units of lysate was transferred to the surface of the gradient after an equal volume was removed from the top of the gradient. Gradients were centrifuged at 41,000 rpm (RCFave  = 207,000) for 70 min at 4°C using a SW41 rotor and then stored at 4°C until fractionation. The Gradient Station (Biocomp) trumpet tip was lowered into the ultracentrifuge tube at a rate of 0.17 mm per second. Fractions (12 drops each, ∼550 µl) were collected into a 96-well plate containing 600 µl of lysis solution (Invitrogen cat.#, with β -mercaptoethanol added) using a fraction collector (Foxy Jr.). The absorbance of the gradient at 260 nm was measured during fractionation using a UV6 system (Teledyne-Isco).

### Gradient Encoding

Gradient Encoding was performed as described previously [29] with changes specified below. Briefly, *in vitro* transcribed mRNAs with 25 nt polyA tails derived from the Methanococcus jannaschii genome were added to each encoded fraction at 100pg per mRNA such that each fraction contained 4-to-6 control mRNAs recognized by 16-to-24 unique probes on the MEEBO DNA microarray (sequences, and PCR primers provided in Table S5). 15 fractions were separated into two pools, A and B, as per Table S6, and the RNA was harvested as per the invitrogen Purelink Micro-to-Midi (since replaced by the Mini cat.# 12183018A) RNA purification kit for liquid samples with the exception that the lysis buffer:lysate:EtOH were in a 1∶1:2 stoichiometry before loading onto the column. 1 ug of purified RNA was amplified using the Amino Allyl MessageAmp II aRNA Kit kit (Ambion cat.# AM1753) and labeled with Cy5 for pool A and Cy3 for pool B. See “Scanning and Data Processing” for assignment of oligos to average ribosome number.

### DNA Microarray Production and Prehybridization Processing

MEEBO oligonucleotide microarrays were printed on epoxysilane-coated glass (Schott Nexterion E) by the Stanford Functional Genomic Facility. The MEEBO microarrays contain ∼39,000 70-mer oligonucleotide probes, representing ∼30,000 unique genes. A detailed description of this probe set can be found at http://www.microarray.org/sfgf/meebo.do. Prior to hybridization, slides were first incubated in a humidity chamber (Sigma Cat# H6644) containing 0.5× SSC (1× SSC  = 150 mM NaCl, 15 mM sodium citrate [pH 7.0]) for 30 min at room temperature. Slides were snap-dried at 70–80°C on an inverted heat block. The free epoxysilane groups were blocked by incubation with 1 M Tris-HCl (pH 9.0), 100 mM ethanolamine, and 0.1% SDS for 20 min at 50°C. Slides were washed twice for 1 min each with 400 ml of water, and then dried by centrifugation. Slides were used the same day.

### DNA Microarray Sample Preparation, Hybridization, and Washing

Amplified RNA was used for all DNA microarray experiments. Poly-adenylated RNAs were amplified in the presence of aminoallyl-UTP with Amino Allyl MessageAmp II aRNA kit (Ambion Cat# 1753). For mRNA expression experiments, universal mouse reference RNA was used as an internal standard to enable reliable comparison of relative transcript levels in multiple samples (Stratagene cat.# 750600). Amplified RNA (5–10 µg) was fluorescently labeled with NHS-monoester Cy5 or Cy3 (GE HealthSciences Cat# RPN5661). Dye-labeled RNA was fragmented (Ambion Cat# 8740), then diluted into in a 50-µl solution containing 3× SSC, 25 mM Hepes-NaOH (pH 7.0), 20 µg of human Cot-1 DNA (Invitrogen Cat# 15279011), 20 µg of poly(A) RNA (Sigma Cat# P9403), 25 µg of yeast tRNA (Invitrogen Cat# 15401029), and 0.3% SDS. The sample was incubated at 70°C for 5 min, spun at 14,000 rpm for 10 min in a microcentrifuge, then hybridized at 65°C using the MAUI hybridization system (BioMicro) for 12–16 h.

For gradient encoding experiments, each fraction was divided into two pools, “A” and “B” according to Table S6. Amplified RNA from pools A was fluorescently labeled with NHS-monoester Cy5, and RNA from pools B was fluorescently labeled with NHS-monoester Cy3. Amplified RNA from pools A and B were comparatively hybridized to a DNA microarray to obtain each mRNA’s average sedimentation within the gradient.

Following hybridization, microarrays were washed in a series of four solutions containing 400 ml of 2× SSC with 0.05% SDS, 20058 SSC, 1× SSC, and 0.2× SSC, respectively. The first wash was performed for 5 min at 65°C. The subsequent washes were performed at room temperature for 2 min each. Following the last wash, the microarrays were dried by centrifugation in a low-ozone environment (<5 ppb) to prevent destruction of Cy dyes. Once dry, the microarrays were kept in a low-ozone environment during storage and scanning (see http://cmgm.stanford.edu/pbrown/protocols/index.html).

### Scanning and Data Processing

Scanning and Data Processing were performed as previously described [29] with the following exceptions. Data were filtered to exclude elements that did not have one of the following: a regression correlation of ≥0.6 between Cy5 and Cy3 signal over the pixels comprising the array element, or an intensity/background ratio of ≥2.5 in at least one channel. For mRNA abundance measurements, red/green ratios were normalized by linear regression to the line fitting the red/green ratios for a set of doping controls (Ambion cat.# AM1780)) doped at various relative concentrations into the total lysate and reference samples for each array (see Table S7 for ratios of Ambion doping controls). The red/green ratio for the encoded samples were related to ribosome number as previously described [29], and as described briefly below. Log_2_(red/green) ratios from the oligos representing the *in vitro* transcribed doping controls were normalized by linear regression to the expected ratios of doping controls from each fraction, allowing empirical red/green ratios to be expressed as a function of fraction number. The mid-point of each fraction was expressed in terms of time and related to the Log of the mid point of each A260 peak expressed in time – allowing fraction number to be expressed in terms of ribosome peaks, and thus red/green ratios to be related to average ribosome numbers.

The Pearson correlations of the log of the average ribosome number between replicate experiments were >0.95 for all untreated and imatinib-treated replicates, and 0.89 to 0.96 for rapamycin replicates. For SAM, unpaired two-class *t*-tests were performed with default settings with mean centering applied to all arrays (R-package samr; http://cran.r-project.org/web/packages/samr/index.html). Only non-control microarray features that passed quality filtering in all replicates were used in the analyses. GO term and KEGG pathway analysis was performed using Gene Trail software [101], which can be found at http://genetrail.bioinf.uni-sb.de/using over-representation analysis with the set of all genes for which translation data were acquired as reference. Settings were all default using the FDR adjustment for multiple testing. The data discussed in this publication have been deposited in NCBI’s Gene Expression Omnibus [102] and are accessible through GEO Series accession number GSE35051 (http://www.ncbi.nlm.nih.gov/geo/query/acc.cgi?acc=GSE35051).

### Proteomics Data Acquisition and Analysis

Imatinib treatment was performed as specified in “Cell Lines and Cell Culture”. Cells were harvested by resuspension in RIPA buffer (25 mM Tris-HCl pH 7.6, 150 mM NaCl, 1% NP-40, 1% sodium deoxycholate, 0.1% SDS), immediately boiled in sample buffer. Lysate from an equal number of medium-labeled, imatinib-treated cells and heavy-labeled, untreated cells (corresponding to roughly 30 µg of each lysate) were run on 4–12% Bis-Tris polyacrylamide gels (BioRad, cat.# 345-0124). The same procedure was carried out for the reverse combination of lysates from heavy-labeled, imatinib-treated and medium-labeled, untreated cells. Each of the two lanes was cut into 8 slices that were interrogated using high-resolution tandem MS using an LTQ OrbiTrap XL mass spectrometer (Thermo-Finnigan) coupled with a nanoLC 2D, a two-dimensional HPLC system (Eksigent). The spectra were acquired in a data-dependent mode in m/z range of 400−1800, with selection of the five most abundant +2 or +3 ions of each MS spectrum for MS/MS analysis. MS/MS spectra were searched using X! Tandem [103] (2007.01.01) with an alternative scoring plugin [104] compatible with PeptideProphet [103]. Searches were conducted against the mouse International Protein Index database (IPI Mouse v3.79) plus common contaminants. All searches used the following parameters: ±2.5 Da precursor mass error, tryptic cleavage with up to one missed cleavage site, static modifications of 57.021 Da (iodoacetamide) on cysteine and 6.0201 Da (medium label) on arginine, potential modifications of 4 Da (difference between medium and heavy label) on arginine, and 15.9949 Da (oxidation) on methionine. Peptide assignments given an identification probability less than 0.95 by PeptideProphet were removed. For each remaining peptide identification, we determined the ratio of medium to heavy isotope, which should reflect the change in production of the corresponding protein after 12 hours of imatinib treatment, using the XPress quantitation algorithm [105]. The quality of the peptide ratio data was ensured as follows: low-quality peptide quantitative ratios were algorithmically identified and removed from the dataset using the previously-described Qurate software tool [106]; all remaining ratios representing a peptide abundance change greater than 1.5-fold in either direction were visually examined using Qurate, and low-quality events were removed; finally, all remaining log-ratios were median-centered on zero to eliminate sample loading bias. Protein inference was performed using ProteinProphet [107]. Since only high-quality peptide identifications were used for protein inference, only proteins with ProteinProphet probability <0.05 were rejected outright. Protein identifications were further restricted to proteins for which at least two unique peptide sequences were identified. Protein quantitative ratios were determined using the geometric mean of all assigned peptide ratios, and the data from both the “forward” and “reverse” experiments were combined (with “reverse” ratios flipped so that all ratios were expressed as treated:untreated) to determine the geometric mean ratios of imatinib-treated/untreated peptides. For comparison to the pSILAC data, translation and mRNA abundance values for multiple oligos representing one gene were averaged (see Table S1).

### Traditional Polysome Profiling

Lysates and sucrose gradients were prepared as in “Translation Profiling” with half the volume (225 uL) collected per fraction. ∼30 pg of a poly-adenylated *M. jannaschii* doping control RNA was added to each fraction prior to harvesting total RNA with the Micro-to-Midi (since replaced by the Mini cat.# 12183018A) RNA isolation kit. An equal volume of RNA (roughly ∼1/3 of the fraction) was reverse-transcribed using Superscript III (Invitrogen, cat.#18080-044) with an oligo(dT)_12-18_ primer (Invitrogen, cat.#18418-12) from each of the fractions. cDNA from each fraction was treated with 1.2 units of RNaseH for 30 minutes at 37°C. Quantitative PCR was performed on 3 µL of cDNA (after diluting them from 1∶5 up to 1∶60 depending on the estimated abundance of the transcript) with inventoried taqman primer and probe sets from Applied Biosystems (see Table S8) using an HT7900 Sequence Detector (ABI). Primer probe sets for taqman for the MJ *in vitro* transcribed RNA are in Table S8. The standard consisted of ten-fold serial dilutions of cDNA made as above from 5 µg of imatinib- treated and untreated total cytoplasmic lysate RNA with 10 pg of control RNA added.

PCR was performed in triplicate on samples, controls and standards; the mean values were divided by the mean values for the doping control reactions with appropriate propagation of error. The values were further normalized such that the sum of the normalized qRT-PCR values for each gradient equaled 1.

The relative abundance of transcripts between treated and untreated samples was calculated by comparing the values of three serial dilutions of untreated to the treated samples. All PCR assays had an R^2^ value of >0.99 for the linear regression of the standards and all values were within the linear range for each assay.

### Explicit Calculation of “Encoding” Values from the qRT-PCR Results

In order to quantitatively validate the encoding method we explicitly “encoded” the values obtained by qRT-PCR for individual fractions using a set of 9 genes with varying SAM scores and local FDR predictions. The mean normalized qRT-PCR value for each fraction was multiplied by the percentage of each fraction that would have been labeled by Cy3 in the encoding experiment. The same analysis was performed for Cy5 for each fraction. The theoretical amounts of Cy3 and Cy5 for each transcript were summed, and the theoretical Cy5/Cy3 ratio for each transcript was calculated. The Cy5/Cy3 ratios were expressed in terms of fractions using the slope and intercept obtained by linear regression of the expected Cy5/Cy3 ratio for each fraction. In order to control for the fact that not all gradients (and the fractions taken therein) are perfectly super-imposable, we expressed the fractions from each gradient in terms of ribosome peaks using the same “peak finding” function in R used for the encoding. As with Gradient Encoding, the Log_2_ ratio of the treated to untreated average ribosome number was calculated. Log_2_ (treated/untreatead) values for genes represented by two oligo probes for the Gradient Encoding were averaged.

## Supporting Information

Figure S1 **qRT-PCR was performed on fractions from high-resolution sucrose gradients on lysates from cells either un-treated (in light blue) or treated with imatinib (in pink).** A) qRT-PCR was performed on an equal portion of RNA harvested from each fraction from the sucrose gradient using ABI inventoried taqman assays for qRT-PCR (see Fig. 2 for the remaining two genes). Values were normalized by those for a doping control RNA added to each fraction prior to harvesting the RNA. The y-axis represents relative absorbance across each gradient (Arbitrary Units, AU). B) The ratio of mRNA abundance in cytoplasmic lysates before and after imatinib treatment was measured in the same experiment using the same taqman probes and primer sets. The untreated samples were normalized to 1 for each individual probe and primer set.
(PDF)

Figure S2 **pSILAC data represents an unbiased set of genes.** The scatterplot of Log_2_ (imatinib-treated/untreated) values for translation vs. mRNA abundance is shown with oligos representing genes represented in the pSILAC data in red.
(PDF)

Figure S3 **Heatmaps of genes in the enriched GO term categories representing the average change upon treatment.** A) Average change in translation (expressed in standard deviations) upon either imatinib- or rapamycin-treatment is depicted from top to bottom in order of increasing change in translation upon rapamycin treatment for the following categories: Translation, Mitochondrial Part (Mitochondria), Antigen Processing, Lysosome, Proteasome, Nucleus, phosphatidylinositol (PI) signaling and regulation of actin cytoskeleton (Actin cytoskeleton). B) The same as A but the change in translation is compared to the change in mRNA abundance upon imatinib treatment in order of increasing change in mRNA abundance. In cases where multiple oligos represent a gene, the oligo with the biggest change in translation upon imatinib treatment was used. Genes that passed the 10% FDR cutoff for translation upon imatinib treatment, but not upon rapamycin treatment (A) or for change in mRNA abundance upon imatinib treatment (B) are highlighted in yellow.
(PDF)

Table S1 **Genes for which we acquired pSILAC, Gradient Encoding, and mRNA abundance data.** Gradient Encoding and mRNA abundance data for multiple oligos representing the same gene were averaged. All values are given as Log_2_ (treated/untreated) and are sorted in ascending order for pSILAC value.
(XLS)

Table S2 **Genes translationally activated by imatinib.** Genes are listed in order of SAM score starting with the most significantly imatinib-regulated gene. Values are given for individual oligos on the array, therefore some genes are represented more than once. Genes with multiple oligos for which the standard deviation between the oligos was more than half the average difference from the mean were assigned footnotes to categorize the nature of the discrepancy between oligos (ie. plausibly due to splice variation, or alternative polyadenylation, etc.). Those genes with discordant oligos that could not be explained by the above criteria (footnote 4) were removed from the bioinformatic functional analyses. The columns indicate the following from left to right: Gene symbol from master dataset, alternate names where MEEBO master dataset gene symbols are absent or possibly misleading, footnote corresponding to the nature of the discrepancy in cases where multiple oligos representing one gene are discordant, accession number, description of the protein product, the Log_2_ ratio of average ribosome number of imatinib-treated to untreated lysate, SAM score, FDR (%), the same for changes in mRNA abundance (Log ratio, SAM, FDR (%)), changes in average ribosome number upon rapamycin treatment (Log ratio, SAM, FDR (%)), and rapamycin-induced changes in mRNA abundance (Log ratio, SAM, FDR (%)), sequence of the oligo probe on the array.
(XLS)

Table S3 **Genes translationally repressed by imatinib in increasing order by SAM score.** Column headings are as in Table S2.
(XLS)

Table S4 **Genes representing the GO term and KEGG pathway enrichments are listed grouped by GO term or KEGG pathway, and subsequently ordered in ascending SAM score of the translational response to imatinib.** Genes belonging to more than one category are listed in each category. Columns as in Table S2.
(XLS)

Table S5 **MJ** ***in vitro*** **transcribed RNA sequences, and PCR primers used to amplify them.**
(XLS)

Table S6 **Pooling of fractions for gradient encoding.**
(XLS)

Table S7 **Ratios of Ambion doping controls used to normalize the mRNA abundance data.**
(XLS)

Table S8 **Taqman probes used for qRT-PCR analysis.**
(XLS)

Data S1 **Master datasheet for all non-control oligos.** Translation change, Abundance change, and pSILAC change are all expressed as Log_2_ (treated/untreated). “Probe” denotes the sequence of the oligo on the MEEBO array to which the data correspond. (Imat.  =  imatinib, Abund.  =  abundance, Rap.  =  rapamycin)
(TXT)
