# Supplementary figures and images for: Extensive Gene-Specific Translational Reprogramming in a Model of B Cell Differentiation and Abl-Dependent Transformation
Source: PLoS One. 2012 May 31;7(5):e37108. doi: 10.1371/journal.pone.0037108 (PMC3365017; doi:10.1371/journal.pone.0037108)

Supplemental Figure 1A

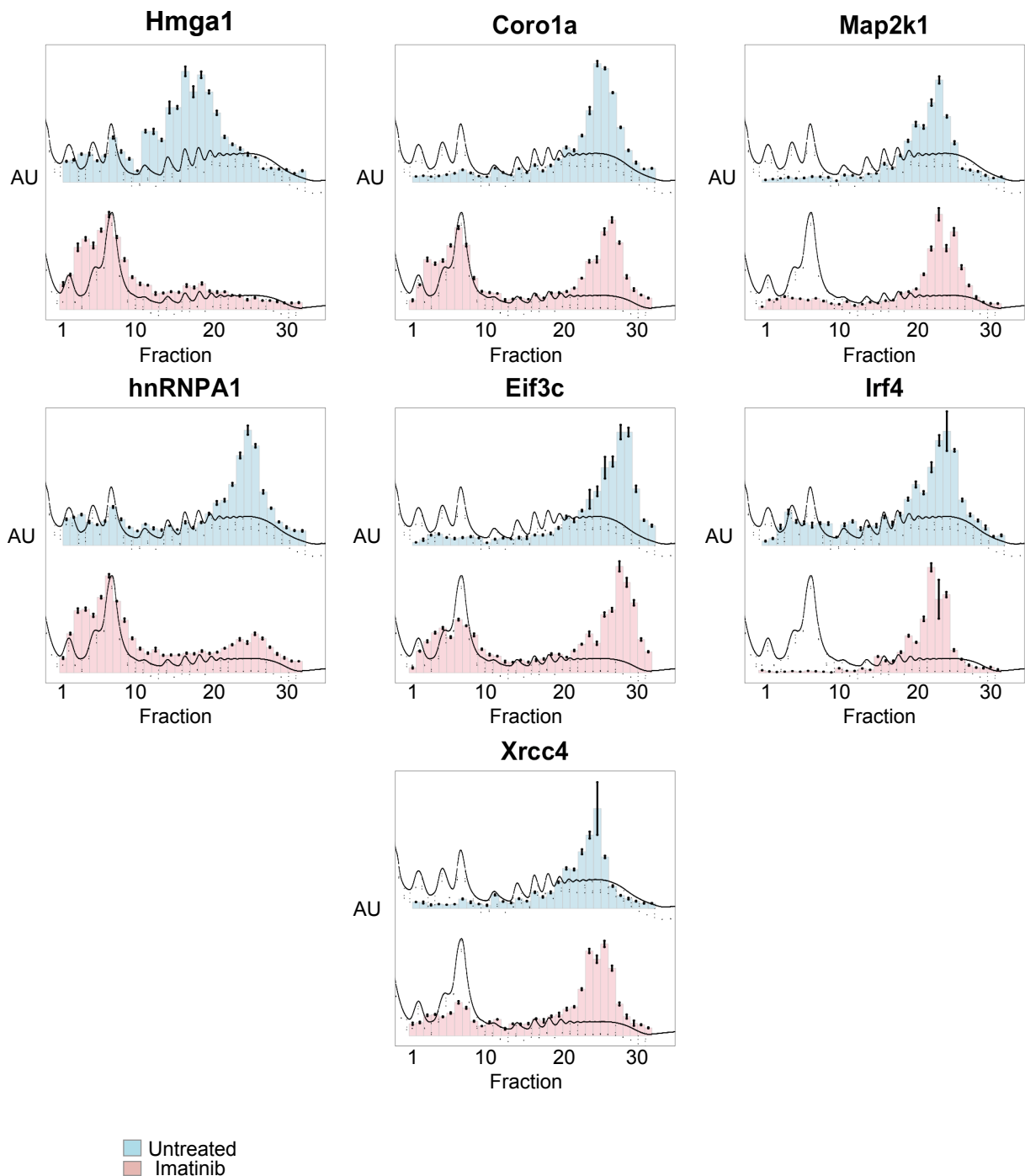

Supplemental Figure 1B

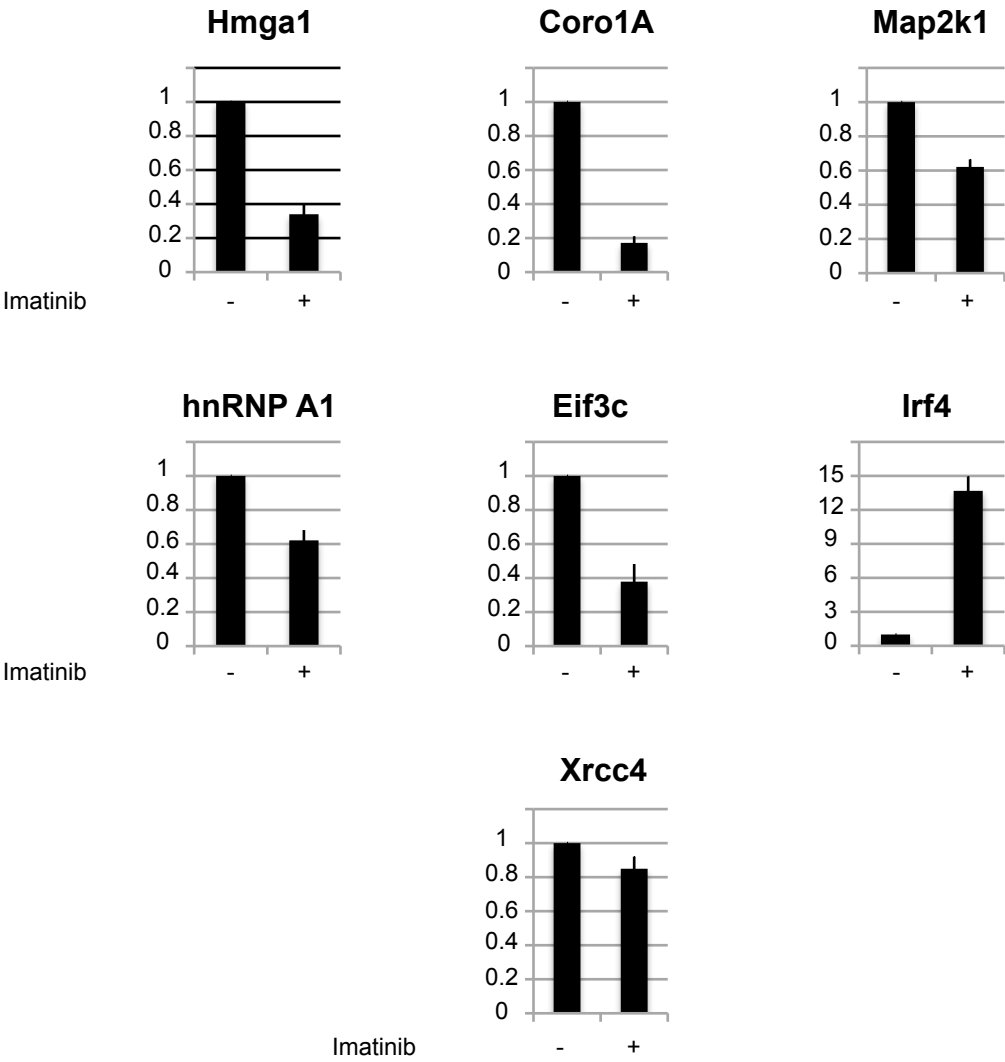

Supplement: Figure S1 — qRT-PCR was performed on fractions from high-resolution sucrose gradients on lysates from cells either un-treated (in light blue) or treated with imatinib (in pink). A) qRT-PCR was performed on an equal portion of RNA harvested from each fraction from the sucrose gradient using ABI inventoried taqman assays for qRT-PCR (see Fig. 2 for the remaining two genes). Values were normalized by those for a doping control RNA added to each fraction prior to harvesting the RNA. The y-axis represents relative absorbance across each gradient (Arbitrary Units, AU). B) The ratio of mRNA abundance in cytoplasmic lysates before and after imatinib treatment was measured in the same experiment using the same taqman probes and primer sets. The untreated samples were normalized to 1 for each individual probe and primer set. (PDF) [file pone.0037108.s001.pdf]

Supplemental Figure 2

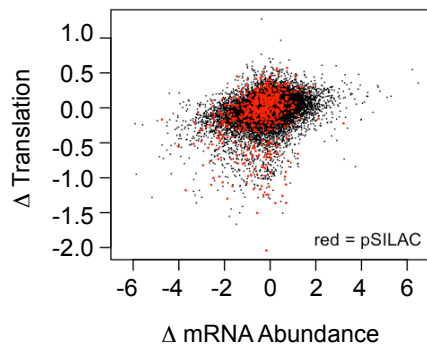

Supplement: Figure S2 — pSILAC data represents an unbiased set of genes. The scatterplot of Log2 (imatinib-treated/untreated) values for translation vs. mRNA abundance is shown with oligos representing genes represented in the pSILAC data in red. (PDF) [file pone.0037108.s002.pdf]

Supplemental Figure 3A

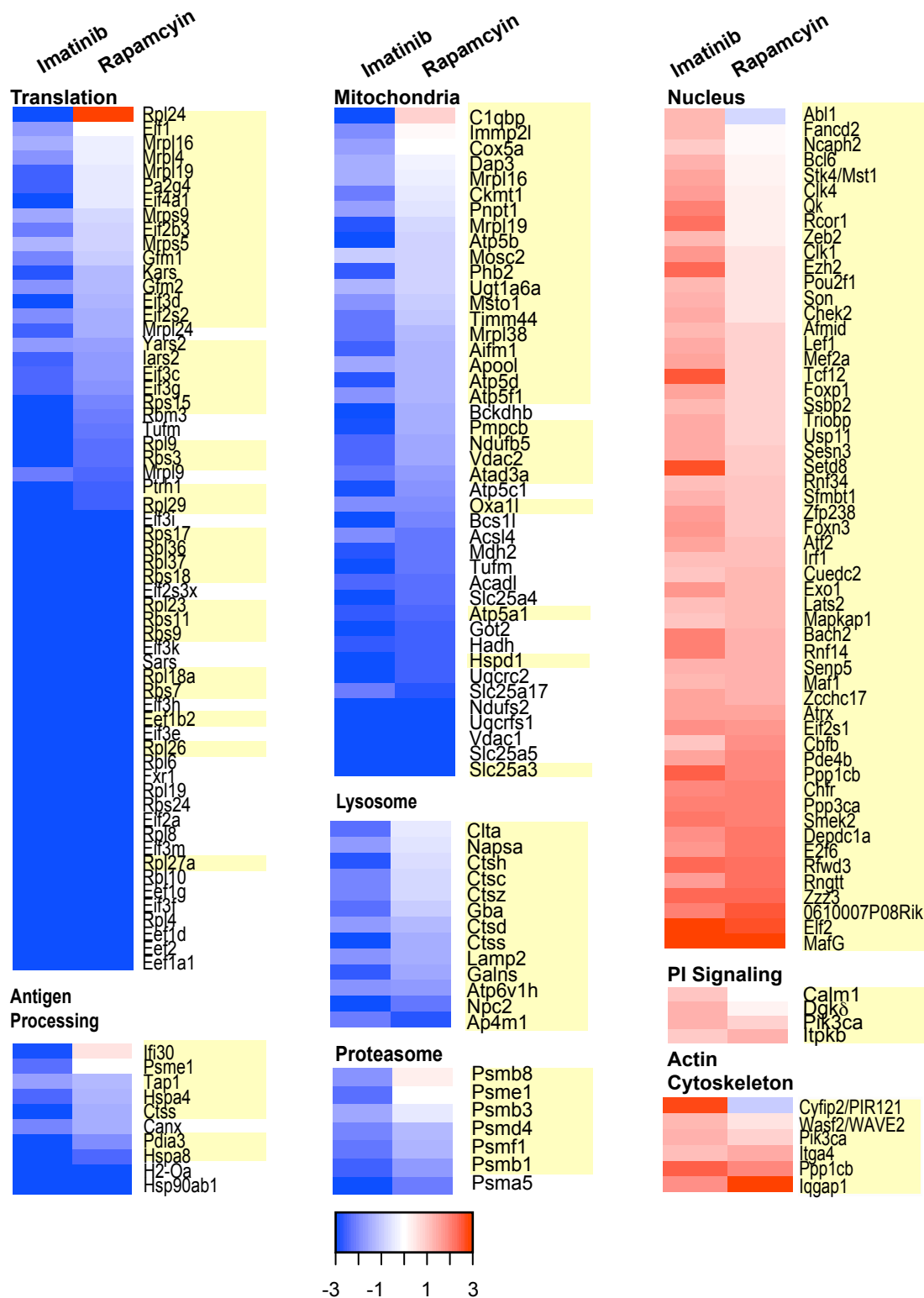

Supplemental Figure 3B

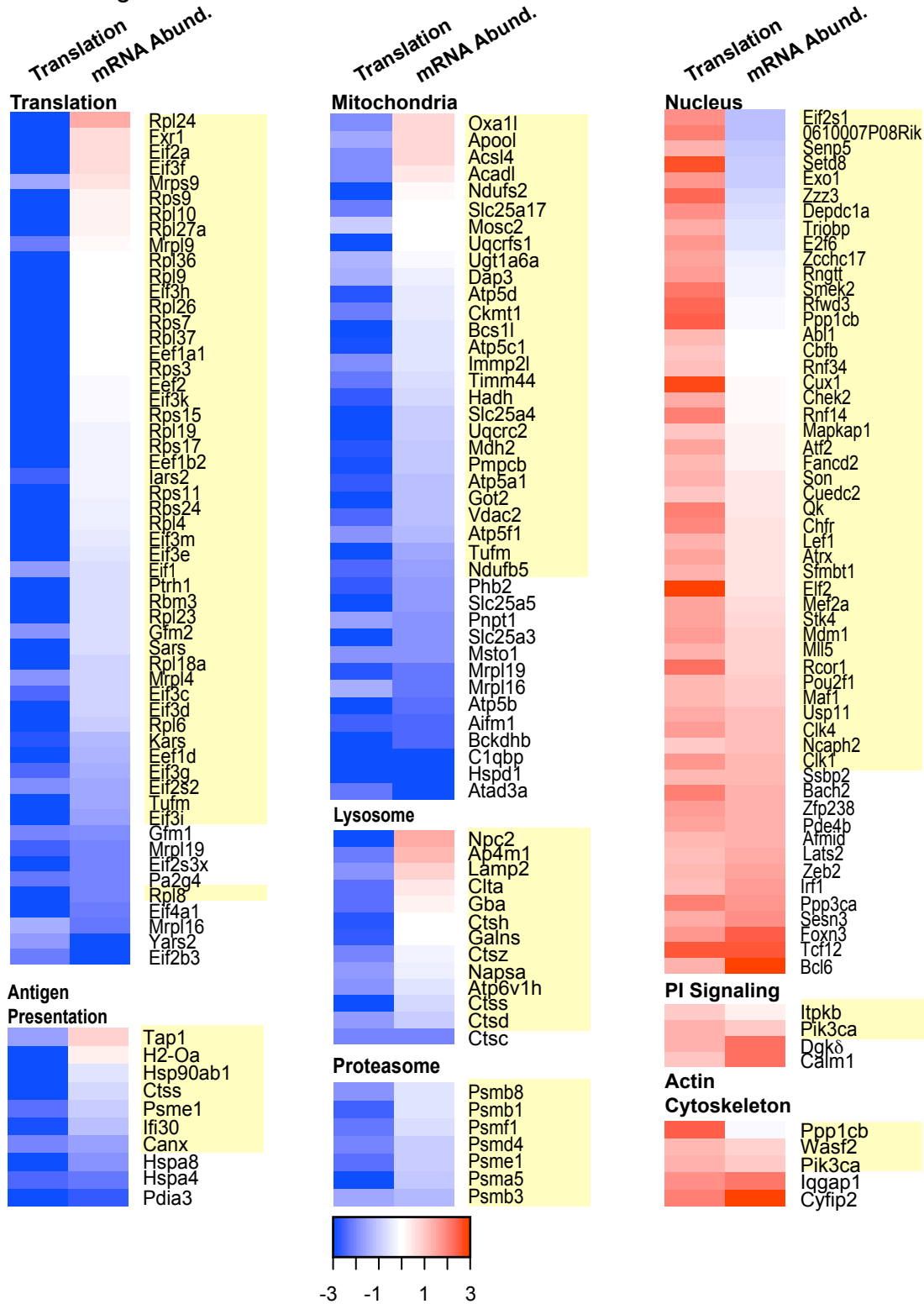

Supplement: Figure S3 — Heatmaps of genes in the enriched GO term categories representing the average change upon treatment. A) Average change in translation (expressed in standard deviations) upon either imatinib- or rapamycin-treatment is depicted from top to bottom in order of increasing change in translation upon rapamycin treatment for the following categories: Translation, Mitochondrial Part (Mitochondria), Antigen Processing, Lysosome, Proteasome, Nucleus, phosphatidylinositol (PI) signaling and regulation of actin cytoskeleton (Actin cytoskeleton). B) The same as A but the change in translation is compared to the change in mRNA abundance upon imatinib treatment in order of increasing change in mRNA abundance. In cases where multiple oligos represent a gene, the oligo with the biggest change in translation upon imatinib treatment was used. Genes that passed the 10% FDR cutoff for translation upon imatinib treatment, but not upon rapamycin treatment (A) or for change in mRNA abundance upon imatinib treatment (B) are highlighted in yellow. (PDF) [file pone.0037108.s003.pdf]
